# Incidence and Factors Associated With Pulmonary Embolism After Upper Extremity Trauma: A Tertiary Hospital Experience in Turkey

**DOI:** 10.7759/cureus.41077

**Published:** 2023-06-28

**Authors:** Volkan Gür, Furkan Yapici, Izzet Özay Subaşı, Mehmet Burak Gökgöz, Mustafa Tosun, Ismail Tardus, Nizamettin Koçkara

**Affiliations:** 1 Orthopedics and Traumatology, Erzincan University Faculty of Medicine, Erzincan, TUR; 2 Pulmonology, Erzincan University Faculty of Medicine, Erzincan, TUR

**Keywords:** upper-extremity fracture, upper-extremity trauma, thromboprophylaxis, central venous catheter (cvc), orthopedics and traumatology, pulmonary embolism, venous thromboembolism (vte)

## Abstract

Introduction

Venous thromboembolism (VTE), particularly pulmonary embolism (PE), is the third highest cause of death in trauma patients who survive beyond the first day. Musculoskeletal surgery is associated with several complications, some of which may be life-threatening, including deep vein thrombosis (DVT) and PE.

Objective

This research aims to describe risk variables for VTE after upper extremity (UE) fracture at a single institution and estimate the incidence of PE following UE fracture.

Methods

The writers accessed the database via their respective universities using the International Standard Classification (ICD) codes. The medical files of patients aged 18 and older who sought treatment at our emergency department for an injury to their UE and also sought treatment at the orthopedics and traumatology clinic between the years 2013 and 2021 were manually scanned. The patients who applied to the Chest Diseases Clinic within 30 days after the trauma and were diagnosed with PE in the ICD code scan were included in the study.

Results

UE trauma was the cause of admission to the emergency department for 3,265 patients, and 21 of those patients (0.64%) were found to have PE. Fifteen of the patients were male, and six were female. The median age was 59 years (IQR 17). There were no deaths associated with PE. One of the patients had a scaphoid fracture, seven patients had a humerus fracture, five patients had a distal radius fracture, two patients had an acromioclavicular joint injury, one patient had a shoulder dislocation, one patient had a finger fracture, four patients had wrist crush injury. Three patients had diabetes mellitus. Five patients were active smokers. JAK-2 gene V617F mutation was detected in one patient. One patient was diagnosed with prostate cancer, and one had gastric cancer. One patient had a central venous catheter. Two patients were being treated for hypothyroidism. Two patients had hypertension.

Conclusion

According to the findings of our research, the probability of developing PE in the days following of an injury to the UE was found to be 0.64%. Patients with UE injuries who are active smokers and who also have diabetes, hypertension, hypothyroidism, cancer, coagulation disorder (JAK2 gene V617F mutation), or a central venous catheter may benefit from anticoagulant prophylaxis. This is because these patients are at a higher risk of developing dangerous blood clots.

## Introduction

Upper-extremity deep vein thrombosis (UE-DVT) is described as the development of a thrombus in the subclavian, axillary, brachial, ulnar, radial, and interosseous veins of the arm's deep venous system. PE has also been reported in up to 36% of UE-DVT patients. About 6% of all DVT cases are UE-DVT, while the rest are lower-extremity deep vein thrombosis (DVT) [1].

Previously, the estimated yearly incidence of UE-DVT was between 0.4 and one per 10,000 people [2]. According to a recent French population-based study, the annual incidence is relatively high at 0.98 per 10,000 [3]. This could translate to a 2.45-fold increase in risk. The incidence of UE-DVT may be on the rise due to the increased use of central venous catheters (CVCs), the prevalence of malignancies, and UE injuries.

UE-DVT can be classified as proximal or distal in anatomy, as well as primary or secondary in nature. Distal UE-DVT refers to ulnar, radial, and interosseous venous thromboses, generally asymptomatic; thus, no further treatment is recommended [4]. More than one vein is implicated in 62% of all instances with proximal UE-DVT: subclavian vein (76%), axillary vein (47%), and brachial vein (36%). The formation of a thrombus may progress proximally in the thorax to the internal jugular (56%) and brachiocephalic (28%) veins.

Primary UE-DVT, also known as, Paget Schroetter syndrome (PSS) is a condition that develops as a result of venous thoracic outlet syndrome, which is rare. Secondary UE-DVT is more prevalent, accounting for 80%-90% of cases. The two most significant risk factors are CVCs (41%) and malignancies (36.3%) [5]. Immobilization, a history of DVT, heart illness, hyper-estrogenic conditions, and recent surgery are other significant risk factors for UE-DVT [6]. There are four main complications of UE-DVT: pulmonary embolism (PE), post-thrombotic syndrome (PTS), recurrence, and mortality. The incidence of symptomatic PE, PTS, and recurrence in UE-DVT patients is 9.2%, 19%, and 4%, respectively [7,8]. The mortality rate of UE-DVT was found to be 6.8%, 29.8%, and 41.6% at 90 days, one year, and three years, respectively [9].

Although contrast venography is the gold standard imaging modality for diagnosing UE-DVT, compression ultrasonography (cUS) is becoming increasingly popular due to its ease of use and non-invasive nature. The sensitivity and specificity of the cUS are 81%-97% and 93%-96%, respectively [10].

PE is a blood clot that obstructs the pulmonary arteries and is most commonly described by Virchow in the 19th century. It induces several pathophysiologic derangements that can lead to significant hemodynamic compromise, particularly if the clot burden is high. The most important determinant of outcomes in PE is the impact on and response of the right ventricle (RV) to the PE. Pulmonary vascular resistance is regulated by several factors, including oxygen-sensing mechanisms, and inflammatory mediators, such as thromboxane A2 and histamine, released in response to clot further increase vasoconstriction [11].

Unlike DVT in the lower extremities, UE-DVT is known to have a benign clinical course; nonetheless, PE might occur. This research aims to estimate the incidence and describe risk factors for PE after upper-extremity trauma-associated venous thromboembolism (VTE) at a tertiary-care institution.

## Materials and methods

**Figure 1:**
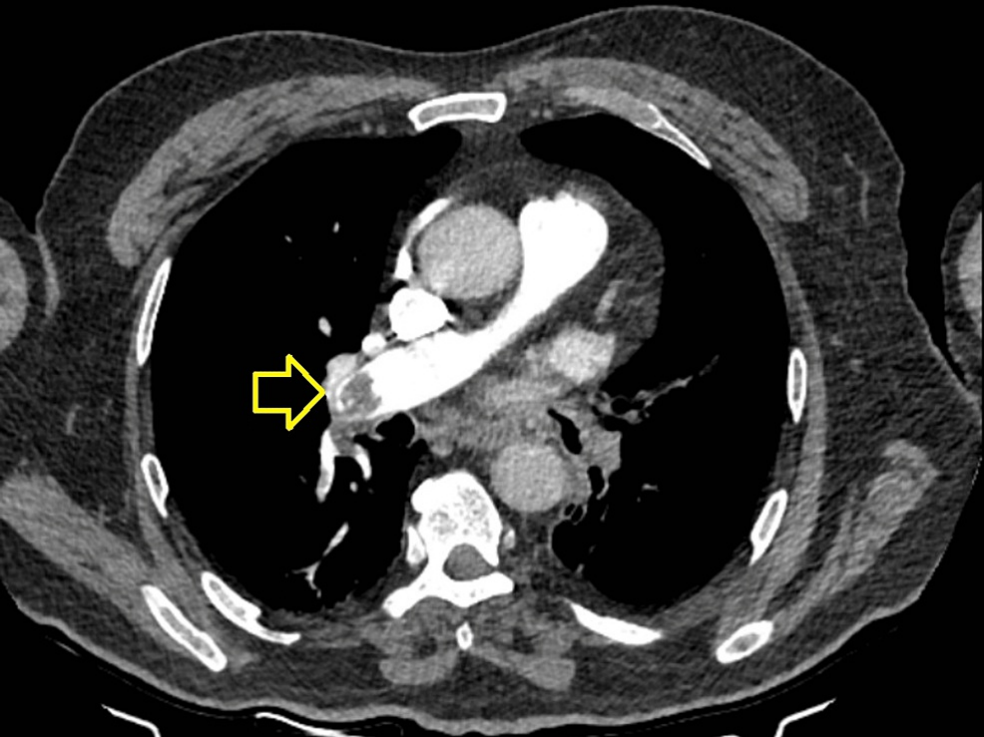
Pulmonary embolism CT view

The authors retrospectively reviewed the patient database of Erzincan University Faculty of Medicine Hospital using International Standard Classification (ICD) codes for upper-extremity trauma, VTE, and PE. The medical files of patients aged 18 and older who sought treatment at our emergency or orthopedics and traumatology department for an upper-extremity injury between 2013 and 2021 were scanned. Patients who presented to the pulmonology or cardiovascular-thoracic surgery department within 30 days following trauma to the UE and were diagnosed with PE were searched. The diagnosis of PE of all patients included in the study was made by a pulmonology specialist or cardiovascular-thoracic surgeon with contrast-enhanced thorax CT (Figure 1). Patients who lacked the necessary data for the research and had an incorrect ICD code or diagnosis were excluded. Demographic data and characteristics of these patients were recorded, including age, gender, smoking history, coagulation disorders, and the presence of a CVC, as well as comorbidities (diabetes, cancer, hypothyroidism, etc.). JAK2 gene V617F mutation was analyzed by real-time PCR method (Ipsogen JAK2 MutaQuant Kit, QIAGEN). This study was approved by the Institutional Review Board of Erzurum Atatürk University Faculty of Medicine. For the treatment of PE, low molecular weight heparin was administered twice daily at a dosage of 1 milligram per kilogram for six months.

## Results

A total of 3,265 patients who applied to Erzincan Binali Yıldırım University Faculty of Medicine emergency service after UE trauma were analyzed retrospectively. Among these patients, 21 patients who applied to the chest diseases outpatient clinic due to dyspnea and were diagnosed with PE by contrast-enhanced thorax CT were included in the study. VTE was detected in 21 patients, and the rate was 0.64%. There were no deaths associated with PE. Fifteen of the patients were male, and six were female. The median age was 59 years (IQR 17). One of the patients had a scaphoid fracture, seven patients had a humerus fracture, five patients had radius distal fractures, two patients had an acromioclavicular joint injury, one patient had shoulder dislocation, one patient had a finger fracture, four patients had a wrist crush injury. Three patients had diabetes mellitus. Five patients were active smokers. JAK-2 gene V617F mutation was detected in one patient. One patient was diagnosed with prostate cancer, and one patient had gastric cancer. One patient had a CVC. Two patients were being treated for hypothyroidism. Two patients had hypertension (Table 1).

**Table 1:** Demographic factors with pulmonary embolism after upper extremity fracture

Patient	Gender(M/F)	Age	Fracture side	Comorbidities
Patient 1	F	75	Humerus fracture	Smoking
Patient 2	M	68	Humerus fracture	Diabetes Mellitus
Patient 3	M	59	Humerus fracture	Smoking
Patient 4	M	83	Humerus fracture	Smoking
Patient 5	M	89	Humerus fracture	Hypertension
Patient 6	M	70	Humerus fracture	Central venous catheter
Patient 7	M	85	Humerus fracture	
Patient 8	M	56	Distal radius fracture	Bleeding disorder
Patient 9	F	60	Distal radius fracture	
Patient 10	M	58	Distal radius fracture	Hypothyroidism
Patient 11	F	83	Distal radius fracture	Smoking
Patient 12	M	34	Distal radius fracture	Diabetes Mellitus
Patient 13	M	56	Wrist-hand crush injury	Smoking
Patient 14	F	53	Wrist-hand crush injury	Malignancy
Patient 15	F	40	Wrist-hand crush injury	Diabetes Mellitus
Patient 16	M	60	Wrist-hand crush injury	Hypothyroidism
Patient 17	M	24	AC seperation	
Patient 18	M	35	AC seperation	
Patient 19	M	57	Phalanx fracture	Hypertension
Patient 20	M	35	Scaphoid fracture	
Patient 21	F	69	Shoulder dislocation	Malignancy

## Discussion

The most important findings of this study were the incidence of PE following UE trauma was 0.64%, UE trauma patients should be actively followed for PE if they are active smokers, have DM, HT, hypothyroidism, malignancy, coagulation disease (JAK2 gene mutation), or a CVC, and anticoagulant prophylaxis may be beneficial for UE trauma patients with these underlying conditions.

VTE and PE are the third leading cause of death in trauma patients who survive beyond the first day [ 12]. Trauma patients are at a greater risk for VTE if they have a prolonged hospital stay, immobilization, extremity fracture, spinal fracture, pelvic fracture, spinal cord injury, or a perceived bleeding risk that contraindicates pharmacologic prophylaxis [13].

Virchow was the first to describe the major causes of venous thrombosis, including vascular endothelium injuries, blood flow alterations, and hypercoagulability. Factors such as a malignant neoplasm, chemotherapy, neurologic disease with paresis, CVC or pacemaker, varicose veins, and superficial vein veins can contribute to the etiogenesis of thrombosis. Congenital or acquired hypercoagulability is found in 20%-30% of DVTs and can be caused by genetic deficiencies of endogen protein C, protein S, and antithrombin III. DVT is also associated with prothrombin gene mutations, anticardiolipin syndrome, lupus anticoagulant, and hyperhomocysteinemia. The occurrence of primary “spontaneous” UE-DVT is infrequent. This condition is characterized by thrombosis of the deep veins that drain the UE. The underlying cause is attributed to anatomical abnormalities of the thoracic outlet, which lead to axillosubclavian compression and consequent thrombosis. The medical condition is commonly known as venous thoracic outlet syndrome, also called PSS and “effort” thrombosis. The condition usually manifests in youthful, asymptomatic individuals as an abrupt and intense discomfort and inflammation in the upper extremities after strenuous UE exertion [14].

Due to the rarity of the disease, the etiology, complications, and clinical outcome of patients with UE-DVT are poorly understood, and the majority of available information comes from case reports or studies with small sample sizes. UE-DVT is a rare thrombotic disorder with potential for considerable morbidity but cannot be diagnosed based on clinical evidence. D-dimer and imaging tests, such as Doppler ultrasonography and CT venography, are not specific indicators of diagnosis. Ultrasonography is a widely available, noninvasive test, but CT venography is less desirable as it is low specificity and time-consuming. A recent review of diagnostic tests found 97% (95% CI, 90-100) sensitivity and 96% (CI, 87-100) specificity for cUS [15].

The most prevalent risk factors for PE were indwelling CVCs and cancer. The PE incidence rate and UE-DVT mortality rate were not low although none of our patients died. Rarely was prevention used. There was no screening protocol or systematic documentation for CVC-associated UE-DVT-at-risk patients. UE-DVT is frequently asymptomatic until complications arise; therefore, it is essential to evaluate risk factors for UE-DVT in order to prevent VTE. Little is known about the clinical outcomes of UE-DVT caused by catheters [16]. A CVC, responsible for between 45% and 72% of all UE-DVT, is the single most critical factor. Additionally, age greater than 77 years, a body mass index of less than 25 kg/m^2^, and hospitalization were independent risk factors for UE-DVT unrelated to a CVC [6].

Current research indicates that 5% of critically ill patients exhibit UE-DVT. Prevention of VTE (prophylaxis) is the standard practice in patients with lower-extremity fractures [17]. Few studies have been conducted on VTE prophylaxis for patients with UE fractures. This may be due to the perception that UE fractures occur less frequently than lower-extremity fractures, or it may be due to a lack of available data with the appropriate sample size and power. Case studies have shown that VTE may occur with fractures of the distal radius, clavicle, and humerus, in addition to ulnar pseudarthrosis [18].

The publication was done by the American College of Chest Physicians its most recent guidelines on thromboprophylaxis for the orthopedic patient in 2012. However, these recommendations were just for individuals who were going to be receiving lower extremity treatments, and they did not include those who were going to be undergoing other types of orthopedic surgeries. For people who just have isolated symptoms, there is no advice UE fractures [19].

Despite the limited research on VTE developing after trauma to the upper extremities, the majority of VTEs an PE developing after fractures of the upper extremities remain asymptomatic [20]. PE is becoming increasingly common due to the rising prevalence of cancer and the usage of indwelling catheters. It is currently uncertain how long treatment should be administered to people with thrombosis caused by cancer. Even after three months of treatment with anticoagulation, the underlying malignancy is generally still active. There is a worry of an increased risk of DVT recurrence in the context of malignancy compared to non-malignancy-associated secondary UE-DVT, even though there is a limited amount of evidence to support this issue. In our study, two patients had a history of malignancy [21].

Our research is limited by the fact that it is retrospective, the vast majority of PEs that occur as a result of UE trauma are asymptomatic, and as a result, the precise number of patients who experience PE as a result of UE trauma is unknown. In addition to this, the small sample size reduces the overall power of the statistical study. We are also aware that regression analysis is required to give more accurate results. Unfortunately, we need more patients for regression analysis. It is possible to achieve this only by doing a multi-center study, which is not possible in the short term. If this study is considered the first study on this subject, it will be a guide for future studies.

## Conclusions

According to the findings of our study, the incidence of PE following trauma to the UE was 0.64%. Anticoagulant prophylaxis might be beneficial for patients with UE trauma who are active smokers and have diabetes, hypertension, hypothyroidism, malignancy, coagulation disease (JAK2 mutation), or a CVC. In addition, the efficacy of prophylaxis for the prevention of UE-DVT or PE remains controversial, with a substantial disparity between national consensus conference panel recommendations and individual practice. In order to improve patient safety during UE-DVT treatment, future research should focus on these issues.

## References

[1] Cote LP, Greenberg S, Caprini JA (2017). Comparisons between upper and lower extremity deep vein thrombosis: a review of the RIETE registry. Clin Appl Thromb.

[2] Grant JD, Stevens SM, Woller SC (2012). Diagnosis and management of upper extremity deep-vein thrombosis in adults. Thromb Haemost.

[3] Delluc A, Le Mao R, Tromeur C (2019). Incidence of upper-extremity deep vein thrombosis in western France: a community-based study. Haematologica.

[4] Yüce G, Bakılan F, Biçen AÇ (2016). Radial vein thrombosis, after radial head fracture: a case report. Türk Osteoporoz Derg.

[5] Illig KA, Doyle AJ (2010). A comprehensive review of Paget-Schroetter syndrome. J Vasc Surg.

[6] Joffe HV, Kucher N, Tapson VF, Goldhaber SZ (2004). Upper-extremity deep vein thrombosis: a prospective registry of 592 patients. Circulation.

[7] Thiyagarajah K, Ellingwood L, Endres K, Hegazi A, Radford J, Iansavitchene A, Lazo-Langner A (2019). Post-thrombotic syndrome and recurrent thromboembolism in patients with upper extremity deep vein thrombosis: a systematic review and meta-analysis. Thromb Res.

[8] Lechner D, Wiener C, Weltermann A, Eischer L, Eichinger S, Kyrle PA (2008). Comparison between idiopathic deep vein thrombosis of the upper and lower extremity regarding risk factors and recurrence. J Thromb Haemost.

[9] Yamashita Y, Morimoto T, Amano H (2019). Deep vein thrombosis in upper extremities: clinical characteristics, management strategies and long-term outcomes from the COMMAND VTE Registry. Thromb Res.

[10] Di Nisio M, Van Sluis GL, Bossuyt PM, Büller HR, Porreca E, Rutjes AW (2010). Accuracy of diagnostic tests for clinically suspected upper extremity deep vein thrombosis: a systematic review. J Thromb Haemost.

[11] Essien EO, Rali P, Mathai SC (2019). Pulmonary embolism. Med Clin North Am.

[12] Knudson MM, Ikossi DG, Khaw L, Morabito D, Speetzen LS (2004). Thromboembolism after trauma: an analysis of 1602 episodes from the American College of Surgeons National Trauma Data Bank. Ann Surg.

[13] Toker S, Hak DJ, Morgan SJ (2011). Deep vein thrombosis prophylaxis in trauma patients. Thrombosis.

[14] Li Y, Wang Z, Sang X (2019). Subclavian vein thrombosis and fatal pulmonary embolism after proximal humerus fracture surgery. J Orthop Surg (Hong Kong).

[15] Lee JA, Zierler BK, Zierler RE (2012). The risk factors and clinical outcomes of upper extremity deep vein thrombosis. Vasc Endovascular Surg.

[16] Claes T, Debeer P, Bellemans J, Claes T (2010). Deep venous thrombosis of the axillary and subclavian vein after osteosynthesis of a midshaft clavicular fracture: a case report. Am J Sports Med.

[17] Ascher E, Salles-Cunha S, Hingorani A (2005). Morbidity and mortality associated with internal jugular vein thromboses. Vasc Endovascular Surg.

[18] Peivandi MT, Nazemian Z (2011). Clavicular fracture and upper-extremity deep venous thrombosis. Orthopedics.

[19] Falck-Ytter Y, Francis CW, Johanson NA (2012). Prevention of VTE in orthopedic surgery patients: antithrombotic therapy and prevention of thrombosis, 9th ed: American College of Chest Physicians Evidence-Based Clinical Practice Guidelines. Chest.

[20] Abelseth G, Buckley RE, Pineo GE, Hull R, Rose MS (1996). Incidence of deep-vein thrombosis in patients with fractures of the lower extremity distal to the hip. J Orthop Trauma.

[21] ALindi SY, Chai-Adisaksopha C, Cheah M, Linkins LA (2018). Management of cancer-associated upper extremity deep vein thrombosis with and without venous catheters at a tertiary care center. Thromb Res.

